# Metabolomics- and proteomics-based multi-omics integration reveals early metabolite alterations in sepsis-associated acute kidney injury

**DOI:** 10.1186/s12916-025-03920-7

**Published:** 2025-02-11

**Authors:** Pengfei Huang, Yanqi Liu, Yue Li, Yu Xin, Chuanchuan Nan, Yinghao Luo, Yating Feng, Nana Jin, Yahui Peng, Dawei Wang, Yang Zhou, Feiyu Luan, Xinran Wang, Xibo Wang, Hongxu Li, Yuxin Zhou, Weiting Zhang, Yuhan Liu, Mengyao Yuan, Yuxin Zhang, Yuchen Song, Yu Xiao, Lifeng Shen, Kaijiang Yu, Mingyan Zhao, Lixin Cheng, Changsong Wang

**Affiliations:** 1 Department of Critical Care Medicine, The First Affiliated Hospital of Harbin Medical University, Harbin, Heilongjiang 150001 China; 2 Department of Critical Care Medicine, First Affiliated Hospital of Southern, Shenzhen People’s Hospital, University of Science and Technology, Shenzhen, 518020 China; 3 Department of Critical Care Medicine, Second Affiliated Hospital of Harbin Medical University, Harbin, Heilongjiang Province 150086 China; 4 Department of Critical Care Medicine, Harbin Medical University Cancer Hospital, Harbin, 150081 China; 5 Heilongjiang Provincial Key Laboratory of Critical Care Medicine, 23 Postal Street, Nangang District, Harbin, Heilongjiang 150001 China

**Keywords:** Sepsis-associated acute kidney injury, Sepsis, Multi-omics analysis, Metabolome, Proteome, Diagnostic biomarkers

## Abstract

**Background:**

Sepsis-associated acute kidney injury (SA-AKI) is a frequent complication in patients with sepsis and is associated with high mortality. Therefore, early recognition of SA-AKI is essential for administering supportive treatment and preventing further damage. This study aimed to identify and validate metabolite biomarkers of SA-AKI to assist in early clinical diagnosis.

**Methods:**

Untargeted renal proteomic and metabolomic analyses were performed on the renal tissues of LPS-induced SA-AKI and sepsis mice. Glomerular filtration rate (GFR) monitoring technology was used to evaluate real-time renal function in mice. To elucidate the distinctive characteristics of SA-AKI, a multi-omics Spearman correlation network was constructed integrating core metabolites, proteins, and renal function. Subsequently, metabolomics analysis was used to explore the dynamic changes of core metabolites in the serum of SA-AKI mice at 0, 8, and 24 h. Finally, a clinical cohort (28 patients with SA-AKI *vs.* 28 patients with sepsis) serum quantitative metabolomic analysis was carried out to build a diagnostic model for SA-AKI via logistic regression (LR).

**Results:**

Thirteen differential renal metabolites and 112 differential renal proteins were identified through a multi-omics study of SA-AKI mice. Subsequently, a multi-omics correlation network was constructed to highlight five core metabolites, i.e., 3-hydroxybutyric acid, 3-hydroxymethylglutaric acid, creatine, myristic acid, and inosine, the early changes of which were then observed via serum time series experiments of SA-AKI mice. The levels of 3-hydroxybutyric acid, 3-hydroxymethylglutaric acid, and creatine increased significantly at 24 h, myristic acid increased at 8 h, while inosine decreased at 8 h. Ultimately, based on the identified core metabolites, we recruited 56 patients and constructed a diagnostic model named IC3, using inosine, creatine, and 3-hydroxybutyric acid, to early identify SA-AKI (AUC = 0.90).

**Conclusions:**

We proposed a blood metabolite model consisting of inosine, creatine, and 3-hydroxybutyric acid for the early screening of SA-AKI. Future studies will observe the performance of these metabolites in other clinical populations to evaluate their diagnostic role.

**Supplementary Information:**

The online version contains supplementary material available at 10.1186/s12916-025-03920-7.

## Background

Sepsis is a life-threatening organ dysfunction caused by a dysregulated immune response to infection. Deaths caused by sepsis account for approximately 20% of total global deaths [1, 2]. Approximately one-third of patients with sepsis in the intensive care unit (ICU) develop sepsis-associated acute kidney injury (SA-AKI) [3], and the mortality rate among patients with SA-AKI is as high as 41% [4]. AKI patients experience longer hospitalizations (+ 3.5 days) and higher medical costs (+ $9000) compared to those without renal impairment [5], emphasizing the critical need for early SA-AKI diagnosis.

Currently, the clinical diagnosis of AKI primarily relies on serum creatinine and urine output [6]. However, creatinine levels exhibit significant individual variability, while urine output is influenced by multiple factors such as fluid volume and cardiac function [7]. Moreover, studies have found that plasma neutrophil gelatinase-associated lipocalin (NGAL) is able to predict renal recovery in patients with SA-AKI [8], but the conclusion is not supported by other researches [9, 10]. For instance, Wong et al. discovered NGAL levels can increase in infectious patients without AKI [9]. Johan et al. found no difference in NGAL between patients with and without AKI [10]. Besides, kidney injury molecule-1 (Kim-1) is another biomarker for renal injury [11], but it is less studied in SA-AKI diagnosis. Thus, it is critical to explore new specific biomarkers for the early diagnosis of SA-AKI.

Renal metabolomics sequencing has focused on small molecular substances, identifying specific biomarkers for renal injury [12]. The integration of metabolomics with other high-throughput omics will be more conducive to the research on biomarkers [13, 14]. Moreover, a large number of machine learning algorithms have been developed and widely used to identify diagnostic and predictive biomarkers, such as support vector machine (SVM) and logistic regression [15–17]. For example, Lusczek et al. used machine learning methods to select three metabolites, lactic acid, 1-methylnicoinamide, and glycine, to predict the death of AKI patients [15]. This study employed proteomic and metabolomic techniques to identify biomarkers associated with SA-AKI. The potential of core metabolites as early diagnostic biomarkers for SA-AKI was evaluated through time series of mice serum. Based on the above biomarkers, a diagnostic model for SA-AKI was constructed within a clinical cohort using machine learning techniques.

## Methods

### Study design

**Fig. 1:**
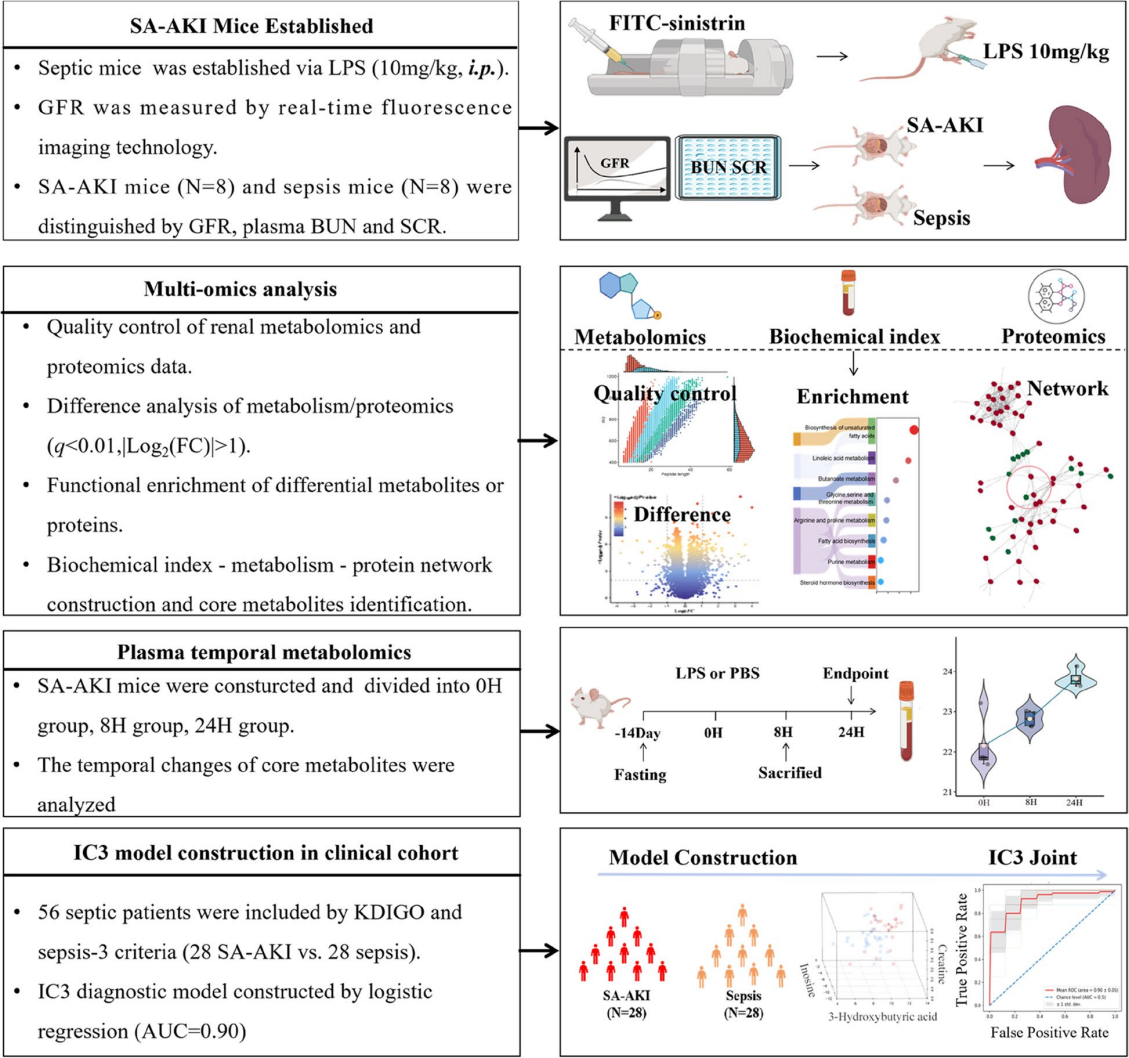
Research design. Biotechnology, bioinformatics, and machine learning methods were integrated in this study to identify effective biomarkers for early diagnosis of SA-AKI. Firstly, SA-AKI and sepsis mice were established through LPS injection, with glomerular filtration rate measured via real-time fluorescence imaging. A Spearman correlation network was constructed from renal metabolomics, proteomics, and biochemical markers in SA-AKI and sepsis mice, from which five core metabolites were identified. Subsequently, we characterized the time series of core metabolites at 0, 8, and 24 h in septic mice to explore their potential as biomarkers for early diagnosis of SA-AKI. Finally, the IC3 model was established based on three core metabolites from a clinical cohort for early prediction of SA-AKI

In this study, we aimed to identify biomarkers for early diagnosis of SA-AKI via multi-omics and clinical data (Fig. 1). Real-time fluorescence imaging technology was utilized to measure the glomerular filtration rate (GFR) [18, 19], combined with assessment of creatinine and blood urea nitrogen (BUN) to distinguish septic mice and SA-AKI mice. Then, we constructed an integrated Spearman correlation network for renal metabolomics and proteomics to highlight five core metabolites, i.e., 3-hydroxybutyric acid, 3-hydroxymethylglutaric acid, inosine, myristic acid, and creatine. The potential of these metabolites as early diagnostic biomarkers was then evaluated by tracking the changes in the activity of five core metabolites in mice serum at three distinct time points: 0, 8, and 24 h. Furthermore, three out of them (inosine, creatine, and 3-hydroxybutyric acid) were validated in a cohort including 56 patients (28 patients with SA-AKI *vs.* 28 patients with sepsis) using targeted quantitative metabolomics. Finally, we developed a logistic regression model to diagnose SA-AKI and to assess the severity of the disease.

### SA-AKI mice construction

Twenty-eight male C57BL/6 mice (aged 8–10 weeks, weighing 25–30 g) were purchased from Charles River Laboratory Animal Technology Co. Ltd. (Beijing, China). The mice were placed in special cages with free access to food and water, four to each cage, and the cages were housed in a room with a 12-h light–dark cycle at a temperature of 22 °C.

Mice were randomly assigned to two parts. In the first part, sixteen mice received LPS (10 mg/kg, *i.p.*) and were euthanized at 24 h. Septic mice or SA-AKI mice were differentiated by measuring glomerular filtration rate (GFR), blood urea nitrogen (BUN), and creatinine levels. In the second part, mice were divided into 3 groups for the experiment, with 4 mice in each group. They were killed at 8 and 24 h following the injection of LPS (10 mg/kg, *i.p.*). The 0-h group (*N* = 4), serving as a control, received an intraperitoneal injection of an equal volume of 0.9% saline. All animal experiments were conducted in compliance with National Institutes of Health guidelines and were approved by the Laboratory Animal Management and Welfare Ethics Committee of the First Affiliated Hospital of Harbin Medical University (Ethical Approval Number: 2023022). The detailed construction process of SA-AKI mice is described in the Additional file 1.

### Patient recruitment and sample collection

From August 2022 to March 2023, a total of 56 patients (28 patients with SA-AKI *vs.* 28 patients with sepsis) were retrospectively enrolled for a clinical study at the Department of Critical Care Medicine, Second Affiliated Hospital of Harbin Medical University. Enrollment criteria included (1) aged between 18 and 65 years old, (2) patients with sepsis meeting the Sepsis-3 criteria [20], and (3) patients with SA-AKI meeting the Kidney Disease: Improving Global Outcomes (KDIGO) diagnostic criteria [21, 22], with an increase in serum creatinine of 0.3 mg/dl (> 26.5 μmol/L) within 48 h, or an increase to more than 1.5 times the baseline value. Exclusion criteria were as follows: (1) discharge or death within 24 h after ICU admission, (2) presence of malignant tumors, (3) immunodeficiency or autoimmune diseases, and (4) insufficient clinical information. In accordance with standardized protocols, all pertinent clinical data were collected.

Peripheral venous blood samples were obtained from patients on the first day of diagnosis. The samples were centrifuged at 3000 rpm for 15 min, and 100 µl of supernatant serum from each sample was immediately frozen in liquid nitrogen and then stored at − 80 °C. The clinical investigation was approved by the Ethics Committee of the Second Affiliated Hospital of Harbin Medical University (Ethical Approval Number: KY2021-188) and strictly complied with the ethical standards of the Declaration of Helsinki.

### Omics research

Detailed metabolomics, proteomic sequencing methods, and data quality control methods are presented in Additional file 1 [23–30].

### Statistical analysis

#### Differential analysis

The values are expressed as mean ± standard deviation (SD) (for continuous variables) or *n* (%) (for categorical variables). Difference analysis was performed using *T*-test (two-sided) on renal metabolomic (8 SA-AKI mice *vs.* 7 sepsis mice) and proteomic data (6 SA-AKI mice *vs.* 6 sepsis mice). The Benjamini–Hochberg correction was applied to control the false discovery rate (FDR), and the significant threshold was set to *q* < 0.01 and |Log_2_(Fold Change)|> 1. Time series serum metabolomics from SA-AKI mice (0 h–8 h–24 h, *N* = 4) were performed via ANOVA test. Mice biochemical indicators (8 SA-AKI mice *vs.* 8 sepsis mice) and human serum targeted quantitative metabolomic sequencing data (28 patients with sepsis *vs.* 28 patients with SA-AKI) were measured by normality test, and then *T*-test (two-sided) or Wilcoxon sign-rank (two-sided) was decided. Serum-targeted quantitative metabolomics sequencing data was then subgroup analyzed by sex, and the significance threshold was set at *p* < 0.05. Univariate logistic regression was applied to evaluate the impact of serum metabolites on SA-AKI, and the odds ratio (OR) was used as a quantitative index to describe the impact.

#### Correlation analysis and network construction

Spearman correlation analysis was used to explore the association between biochemical indicators and differential renal metabolites or proteins, and the significant threshold of the correlation was set at *q* < 0.01. Core metabolites with a metabolite-protein degree > 50 were identified. Multi-omics networks were constructed using Cytoscape (v3.10.0) software to elucidate their interactions and biological functions.

#### Machine learning models

Receiver operating characteristic (ROC) curves were generated to evaluate the diagnostic capability of 3-hydroxybutyric acid, creatine, and inosine in differentiating SA-AKI from patients with sepsis. Clinical cohort data were randomly divided into training (70%) and test (30%) sets. Four machine learning algorithms, including logistic regression (LR), random forest (RF), support vector machine (SVM), and extreme gradient boosting (XGB), were applied to train diagnostic models. Model performance was robustly estimated by calculating the average AUC across 10 independent test sets. Statistical analysis and modeling procedures were performed via RStudio (v4.2.2).

## Results

### Metabolomic variations between SA-AKI and sepsis

**Fig. 2:**
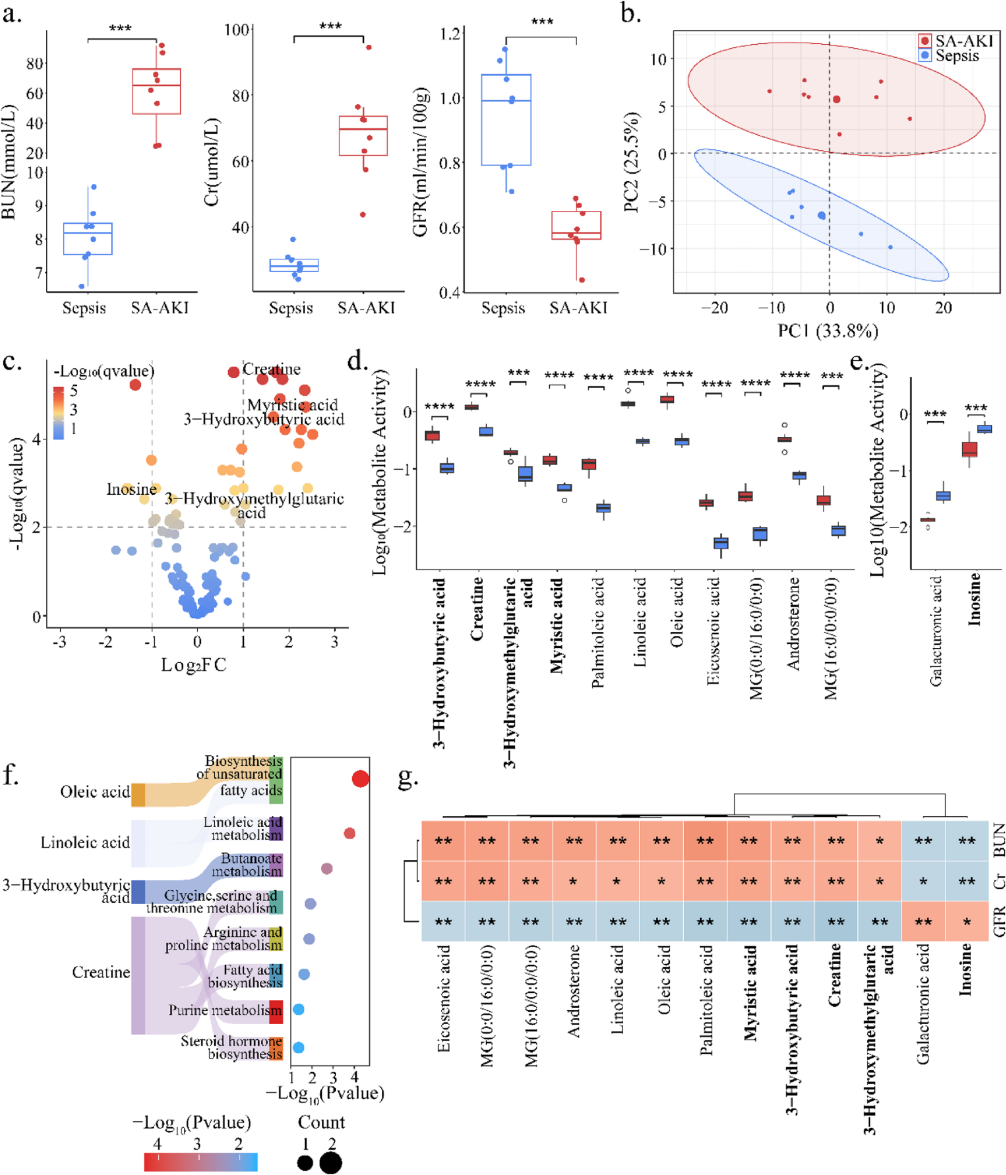
Systematic analysis of metabolic activity differences between SA-AKI and sepsis. **a** Box plots present the biochemical indicators in SA-AKI and sepsis mice, including BUN, creatinine, and GFR. **b** PCA plot of renal metabolomics. Blue points represent the sepsis group (*N* = 7), while red points represent the SA-AKI group (*N* = 8). **c** Differential analysis of renal metabolomics between the two groups. Colors of points correspond to *q*-values. Significant after Benjamini–Hochberg adjusted two-tailed t-test (*q* < 0.01) and |Log_2_(Fold Change)|> 1. **d** Activity distribution of the 11 upregulated metabolites. **e** Activity distribution of the 2 downregulated metabolites. Red represents the SA-AKI group and blue represents the sepsis group. **f** Kegg pathway analysis on renal differential metabolites. **g** Heatmap illustrating the Spearman correlation coefficients between the 13 differential metabolites and the three biochemical indicators. Orange represents a positive correlation and light blue represents a negative correlation. *, **, and *** indicate *q* < 0.05, *q* < 0.01, and *q* < 0.001, respectively

Plasma BUN (65 [46,76] *vs.* 8.19 [7.28,9.1], *p* < 0.05) and creatinine (70 [62,73] *vs.* 28 [26,30], *p* < 0.05) were significantly higher in SA-AKI mice than in sepsis mice, and GFR (0.59 ± 0.08 *vs.* 0.95 ± 0.17, *p* < 0.05) was significantly lower in SA-AKI mice than in sepsis mice (*p* < 0.05) (Fig. 2a; Additional file 2: Table S1). Combining with GFR, BUN, and creatinine indicated that the SA-AKI mice were successfully established [23].

There was a clear change in metabolic activity between SA-AKI mice and sepsis mice (Fig. 2b). The thirteen renal metabolites with significant changes between SA-AKI and sepsis were detailed in Table 1, among which eleven are upregulated and two are downregulated (*q* < 0.01, |Log_2_(FC)|> 1) (Fig. 2d–e; Additional file 2: Table S2–3). The differential metabolites primarily participate in pathways such as biosynthesis of unsaturated fatty acids, linoleic acid metabolism, and butanoate metabolism (Fig. 2f). Among them, linoleic acid is a product of linoleic acid metabolism. Zeng et al. has found that reducing linoleic acid can ameliorate cisplatin-induced AKI [31]. In addition, studies have indicated that linoleic acid breakdown products are associated with acute respiratory distress syndrome and sepsis-related deaths in severe COVID-19 patients [32, 33]. Notably, each differential metabolite was significantly correlated with at least one renal function indicator (*q* < 0.01) (Fig. 2g), suggesting a potential link between changes in renal metabolism and renal impairment.

**Table 1:** Differentially regulated metabolites between SA-AKI and sepsis group

Name	HMDB IDs	Log_2_FC	*q*-values
Palmitoleic acid	HMDB0003229	2.52	< 1e − 04
Eicosenoic acid	HMDB0002231	2.36	< 1e − 04
Oleic acid	HMDB0000207	2.34	< 1e − 05
Linoleic acid	HMDB0000673	2.27	< 1e − 04
MG(0:0/16:0/0:0)	HMDB0011533	2.22	< 1e − 03
Androsterone	HMDB0000031	2.17	< 1e − 03
3-Hydroxybutyric acid	HMDB0000011	1.91	< 1e − 04
MG(16:0/0:0/0:0)	HMDB0011564	1.76	< 1e − 02
Myristic acid	HMDB0000806	1.66	< 1e − 04
Creatine	HMDB0000064	1.42	< 1e − 05
3-Hydroxymethylglutaric acid	HMDB0000355	1.04	< 1e − 02
Inosine	HMDB0000195	− 1.17	< 1e − 02
Galacturonic acid	HMDB0002545	− 1.53	< 1e − 02

Specifically, 3-hydroxybutyric acid (Log_2_(FC) = 1.91) and creatine (Log_2_(FC) = 1.42) were significantly increased, while inosine was significantly decreased in SA-AKI mice (Log_2_(FC) = − 1.17). In addition, both of 3-hydroxybutyric acid and creatine were negatively correlated with GFR (*R* = − 0.80, *R* = − 0.90) while these two metabolites were positively associated with BUN (*R* = 0.73, *R* = 0.69). Inosine was negatively correlated with BUN (*R* = − 0.67). These results highlighted the important effects of these metabolites in SA-AKI mice and revealed potential mechanisms of renal injury.

### Proteomic and metabolomic network analysis identifies core metabolites

**Fig. 3:**
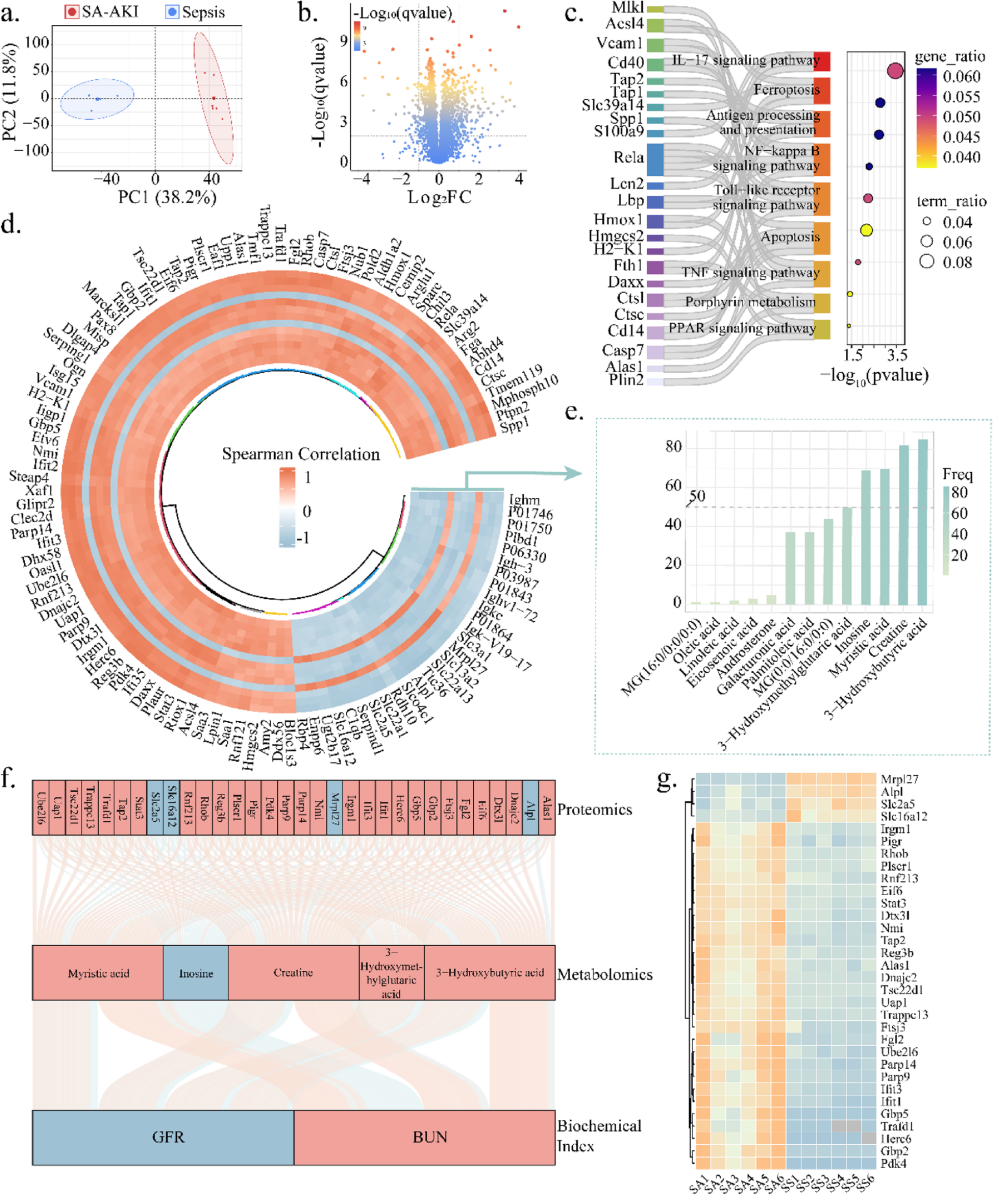
Integrated analysis of mice renal metabolomic and proteomic data. **a** PCA plot of renal proteomics. Blue points represent the sepsis group (*N* = 6), while red points represent the SA-AKI group (*N* = 6). **b** Differential analysis of renal proteomics between the two groups. Colors of points correspond to *q*-values. Significant after Benjamini–Hochberg adjusted two-tailed *t*-test (*q* < 0.01) and |Log_2_(Fold Change)|> 1.** c** KEGG pathway analysis on renal differential proteins. **d** Spearman correlation between differential renal proteins and 16 differential renal metabolites. Orange represents a positive correlation while light blue represents a negative correlation. **e** Degree distribution for the differential metabolites, among which metabolite-protein degree > 50 were defined as core metabolites. **f** Sankey diagram depicting the interrelationships between renal proteins, core renal metabolites, and biochemical indicators. Red lines represent positive correlation, and light blue lines represent negative correlation. Red grid indicates up-regulation and light blue grid indicates down-regulation in SA-AKI mice.** g** Differential proteins related to the five core metabolites

The protein expression significantly changed between SA-AKI mice and sepsis mice (Fig. 3a). Renal proteins significantly dysregulated in SA-AKI mice compared with sepsis (*q* < 0.01, |Log_2_ (FC)|> 1), with 122 being up-regulated and 42 down-regulated (Fig. 3b; Additional file 3: Table S1). 112 differential proteins associated with renal function were detected for further analysis (*q* < 0.01) (Additional file 3: Fig. S1b).

KEGG pathway analysis showed that renal differential proteins were mainly involved in inflammatory pathways such as NF-kappa B signaling pathway, IL-17 signaling pathway, and Toll-like receptor signaling pathway*.* Activation of cell death pathways such as adipocytokine signaling pathway, ferroptosis, and apoptosis pathway were also found (Fig. 2c). These may be related to the impaired mitochondrial function during the occurrence of SA-AKI [14, 34]. Gene enrichment analysis found that biological process (BP) was mainly enriched in reactive oxygen species biosynthetic process, iron ion transmembrane transport, macrophage activation, and regulation of lipid metabolic process (Additional file 3: Fig. S1c), cell composition (CC) was enriched in lipoprotein particle, high-density lipoprotein particle, lipid droplet (Additional file 3: Fig. S1d), molecular functional (MF) process was enriched in oxidoreductase activity, acting on metal ions and lipid transporter activity (Additional file 3: Fig. S1e). Abnormal lipid metabolism can trigger an inflammatory response that promotes cell death [35], leading to a poor prognosis for AKI [36].

Renal metabolomic and proteomic expressions were strongly correlated, for instance, consistent change was found between 3-hydroxybutyric acid and Mphosph10 in our research (cor > 0.99,*q* < 0.01) (Fig. 3c). 3-Hydroxymethylglutaric acid, 3-hydroxybutyric acid, creatine, myristic acid, and inosine were considered as core metabolites of SA-AKI, with a metabolite-protein connectivity threshold of 50 (Fig. 3d). Significant associations (*q* < 0.01) were found between 32 proteins, 5 core metabolites, and renal function indicators, highlighting the continuous interaction between multi-omics within the SA-AKI renal system (Fig. 3e). The 28 upregulated and 4 downregulated proteins may be linked to the function of core metabolites, implicated in the regulation of immune response (Fig. 3f–g; Additional file 3: Fig. S1c–e).

**Fig. 4:**
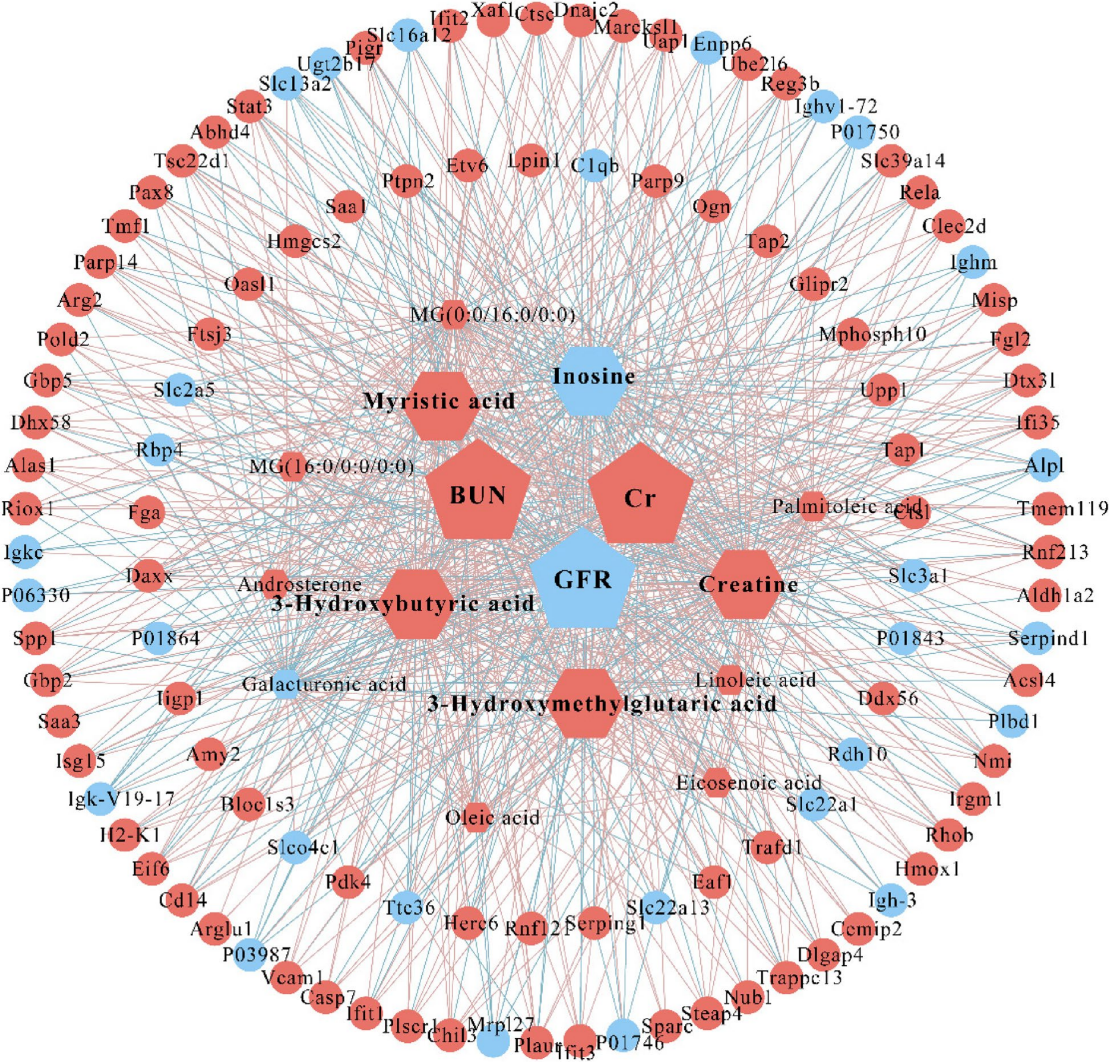
Multi-omics network of SA-AKI. Proteins and metabolites correlated to the core metabolites and biochemical indicators are shown in the network. Biochemical indicators, core metabolites, and renal proteins are represented by pentagon, hexagon, and circle, respectively. Red vertex indicates up-regulation and blue vertex indicates down-regulation between SA-AKI and sepsis groups. Red edge represents a positive correlation and blue edge represents a negative correlation between the vertexes

Five core metabolites, along with 112 differential renal proteins and 13 differential renal metabolites, were involved in regulating changes in renal function through network analysis (Fig. 4). Core metabolites may play a crucial role in the progression of sepsis to SA-AKI, which may provide potential strategies for clinical diagnosis.

### Serum time series investigation of core metabolites in SA-AKI mice

**Fig. 5:**
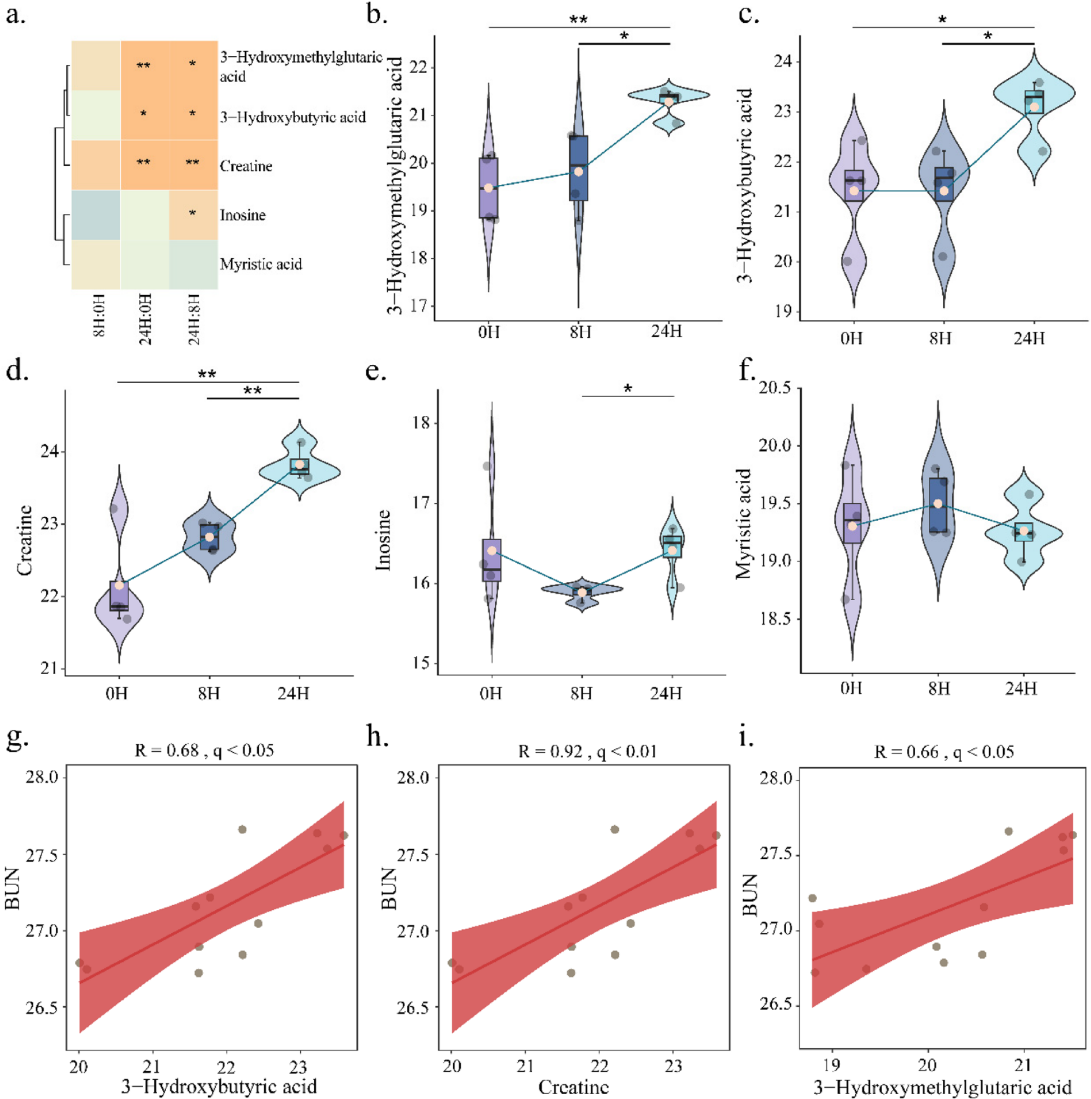
Time series observation of serum metabolites in SA-AKI mice. Changes in serum metabolites in SA-AKI mice at different time points (0H, 8H, and 24H) (**a**–**f**). **a**,** b**,** c**,** d**,** e**, and** f** show the time variation of serum 3-hydroxymethylglutaric acid, 3-hydroxybutyric acid, creatine, inosine, and myristic acid, respectively. **g**–**i** Scatter plots illustrating the correlation between 3-hydroxybutyric acid, creatine, and BUN. *, *p* < 0.05; **, *p* < 0.01

We performed serum metabolomic analyses of SA-AKI mice at three time points (0 h, 8 h, 24 h) to investigate the triggering time of the core metabolites (Fig. 5a; Additional file 4: Table S1). The metabolic activities of 3-hydroxymethylglutaric acid, creatine, and 3-hydroxymethylglutaric acid changed significantly from 0 to 8 h to 24 h (*p* < 0.05, ANOVA test), while inosine and myristic acid remained stable (*p* > 0.05, ANOVA test). Specifically, the metabolic activity of 3-hydroxymethylglutaric acid, 3-hydroxybutyric acid, and creatine significantly increased from 0 to 24 h and from 8 to 24 h (*p* < 0.05) (Fig. 5b–d). Inosine showed a descent trend from 0 to 8 h, while it showed an increasing change from 8 to 24 h (*p* < 0.05) (Fig. 5e). Myristic acid showed an increasing trend at 0 to 8 h and a decreasing trend at 8 to 24 h (Fig. 5f).

We further explored the dynamic tendency between serum core metabolites and blood urea nitrogen (BUN). 3-Hydroxybutyric acid, creatine, and 3-hydroxymethylglutaric acid demonstrated a positive correlation with BUN (3-hydroxybutyric acid: *R* = 0.68, *q* < 0.05; creatine: *R* = 0.92, *q* < 0.01; 3-hydroxymethylglutaric acid: *R* = 0.66, *q* < 0.05) (Fig. 5g–i). Furthermore, no significant correlations were found between inosine, myristic acid, and BUN (Additional file 4: Fig. S1a–b).

### Construction of IC3 model via targeted quantitative serum metabolomics

A clinical study involving 56 patients (28 patients with sepsis *vs.* 28 patients with SA-AKI) was conducted to verify the diagnostic efficacy of the IC3 model with a balanced gender ratio (30 were male and 26 were female). The 30 male patients consist of 16 patients with SA-AKI and 14 patients with sepsis. For the female patients, 12 patients with SA-AKI and 14 patients with sepsis are included (Table 2). We found patients with SA-AKI showed increased levels of creatinine, urea, total and direct bilirubin, along with higher SOFA and APACHE II scores (*p* < 0.05). They also had significantly lower diastolic blood pressure and reduced platelet counts (*p* < 0.05) (Table 2).

**Table 2:** Baseline characteristics of sepsis and SA-AKI patients

	Sepsis group (*n* = 28)	SA-AKI group (*n* = 28)	*P* value
Age	54 ± 16	50 ± 14	0.388
Sex			0.592
Male	14 (50.0%)	16 (57.1%)	
Female	14 (50.0%)	12 (42.9%)	
HR (bpm), median, (IQR)	121 (95, 133)	105 (95, 121)	0.475
SBP (mmHg), median, (IQR)	122 (104, 153)	101 (81, 132)	0.054
DBP (mmHg), median, (IQR)	70 (60, 86)	56 (49, 67)	0.011
MAP (mmHg), median, (IQR)	88 (75, 106)	69 (61, 89)	0.029
RR (bpm), median, (IQR)	22 (17, 35)	23 (18, 28)	0.407
Temperature (°C), median, (IQR)	36.50 (36.50, 36.53)	37.05 (36.50, 38.20)	0.012
SOFA, median, (IQR)	7.00 (6.00, 8.00)	9.00 (8.75, 10.00)	< 0.001
APACHE II, median, (IQR)	15.0 (14.0, 16.0)	22.0 (20.0, 24.0)	< 0.001
WBC (10^9/l), median, (IQR)	15 (10, 29)	13 (9, 17)	0.210
Neutrophil (10^9/l), median, (IQR)	14 (9, 28)	12 (7, 15)	0.321
Monocyte (10^9/l), median, (IQR)	0.33 (0.21, 0.49)	0.30 (0.16, 0.36)	0.426
Lymphocyte (10^9/l), median, (IQR)	1.12 (0.88, 1.62)	0.79 (0.57, 0.99)	0.012
Platelet (10^9/l), mean ± SD	261 ± 89	152 ± 123	< 0.001
PH, median, (IQR)	7.26 (7.22, 7.43)	7.36 (7.28, 7.47)	0.059
HCO3^−^ (mmol/L), mean ± SD	20 ± 7	18 ± 6	0.161
Glucose (mmol/L), median, (IQR)	8 (7, 12)	8 (6, 9)	0.192
Lac (mmol/L), median, (IQR)	3.10 (1.80, 5.35)	2.90 (1.30, 4.80)	0.545
PCT (ng/mL), median, (IQR)	22 (2, 36)	33 (24, 46)	0.083
ALT (U/L), median, (IQR)	17 (11, 55)	17 (11, 73)	> 0.999
AST (U/L), median, (IQR)	32 (24, 87)	33 (27, 85)	0.928
Creatinine (μmol/L), median, (IQR)	117 (65, 182)	263 (158, 476)	< 0.001
Urea (mg/dl), median, (IQR)	9 (5, 15)	26 (16, 31)	< 0.001
TBIL (μmol/L), median, (IQR)	17 (7, 23)	40 (22, 84)	< 0.001
DBIL (μmol/L), median, (IQR)	7 (5, 12)	34 (13, 55)	< 0.001
IBIL (μmol/L), median, (IQR)	6 (2, 10)	11 (7, 16)	0.008

*HR* heart rate, *BPM* beats per minute, *SBP* systolic blood pressure, *DBP* diastolic blood pressure, *MAP* mean arterial pressure, *RR* respiration rate, *SOFA* Sequential Organ Failure Assessment, *APACHE II* Acute Physiological and Chronic Health Assessment, *Lac* lactic acid, *PCT* procalcitonin, *ALT* alanine aminotransferase, *AST* aspartate aminotransferase, *TBIL* total bilirubin, *DBIL* direct bilirubin, *IBIL* indirect bilirubin

**Fig. 6:**
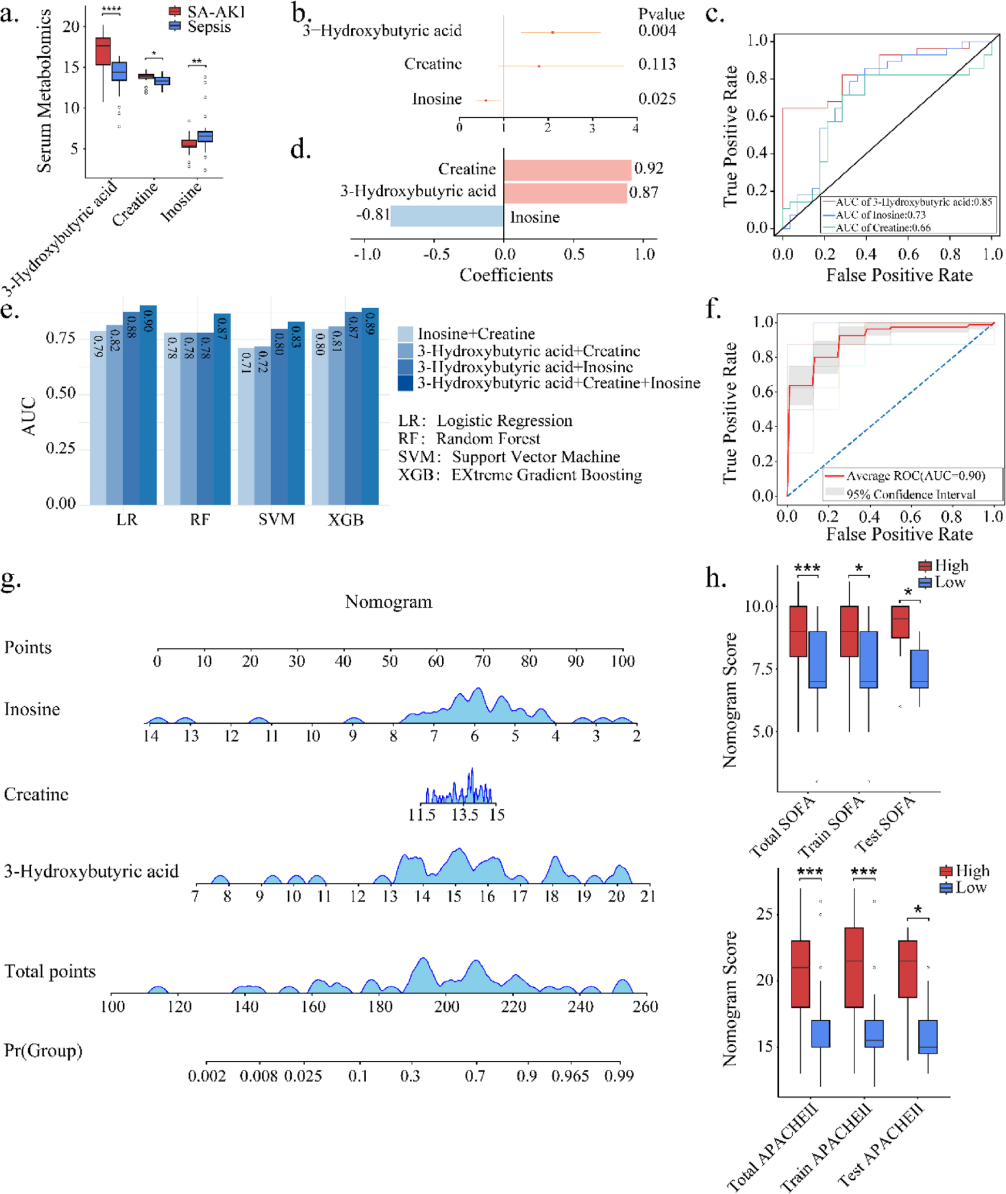
Construction and Validation of the IC3 Model. **a** Abundance distribution of serum metabolites between the SA-AKI (*N* = 28) group and the sepsis group (*N* = 28). (Significant after Wilcoxon univariate testing (*p* < 0.05)). **b** Forest plot showing univariate logistic regression results of three metabolites. **c** ROC curves illustrating the three serum metabolites in diagnosing patients with SA-AKI. **d** Weights of the three serum metabolites in the LR model. **e** The average AUC values for different combinations of three serum metabolites in the diagnosis of SA-AKI via four machine learning methods. **f** Average AUC curve of IC3 model using the three core metabolites. **g** IC3 nomogram. **h** Distributions of SOFA and APACHE II scores for the high-risk (red) and low-risk (blue) patients, which were stratified by the median of the IC3 score. The distributions of the total sample, training set, and test set were separately shown. (Significant after two-tailed *t*-testing (*p* < 0.05)). *, **, and *** indicate *p* < 0.05, *p* < 0.01, and *p* < 0.001, respectively

Three core metabolites were completely detected among the 56 patients by the targeted quantitative metabolomics (Additional file 4: Table S2). The metabolite level of 3-hydroxybutyric acid (Log_2_(FC) = 3.25, *p* < 0.05) and creatine (Log_2_(FC) = 0.33, *p* < 0.05) were increased, while the level of inosine was decreased in SA-AKI (Log_2_(FC) = − 4.06, *p* < 0.05) (Fig. 6a). In the male patient group, 3-hydroxybutyric acid (Log_2_(FC) = 3.19, *p* < 0.01) and creatine were significantly increased (Log_2_(FC) = 0.68, *p* < 0.01) while inosine was significantly decreased (Log_2_(FC) = − 4.64, *p* < 0.05) in patients with SA-AKI (Additional file 4: Fig. S1c–f). In the female patient group, 3-hydroxybutyric acid was significantly increased (Log_2_(FC) = 8.77, *p* < 0.001) while inosine was significantly decreased (Log_2_(FC) = 0.57, *p* < 0.05), but no significant change was observed for creatine in patients with SA-AKI (Additional file 4: Fig. S1c, Fig. S1g–i). To evaluate the effect of three core metabolites on SA-AKI, odds ratio (OR) was 2.10 for 3-hydroxybutyric acid (*p* < 0.05), 1.79 for creatine (*p* > 0.05), and 0.59 for inosine (*p* < 0.05) (Fig. 6b). 3-Hydroxybutyric acid demonstrated higher diagnostic accuracy for SA-AKI (AUC = 0.85) in comparison to inosine (AUC = 0.73) and creatine (AUC = 0.66) (Fig. 6c).

When integrating the three metabolites using the logistic regression (LR) model, it exhibits a much higher predictive performance (AUC = 0.90) (Fig. 6e–f). The average coefficients for 3-hydroxybutyric acid, creatine, and inosine were 0.87, 0.92, and − 0.81, respectively (Fig. 6d). Moreover, an IC3 nomogram was created for a detailed assessment of each sample (Fig. 6g). Samples were categorized into high or low score groups based on the median score. SOFA and APACHE II scores were significantly higher in the high-risk group compared to the low-risk group (*p* < 0.05) (Fig. 6h), demonstrating that IC3 is also an indicator for disease severity.

## Discussion

**Fig. 7:**
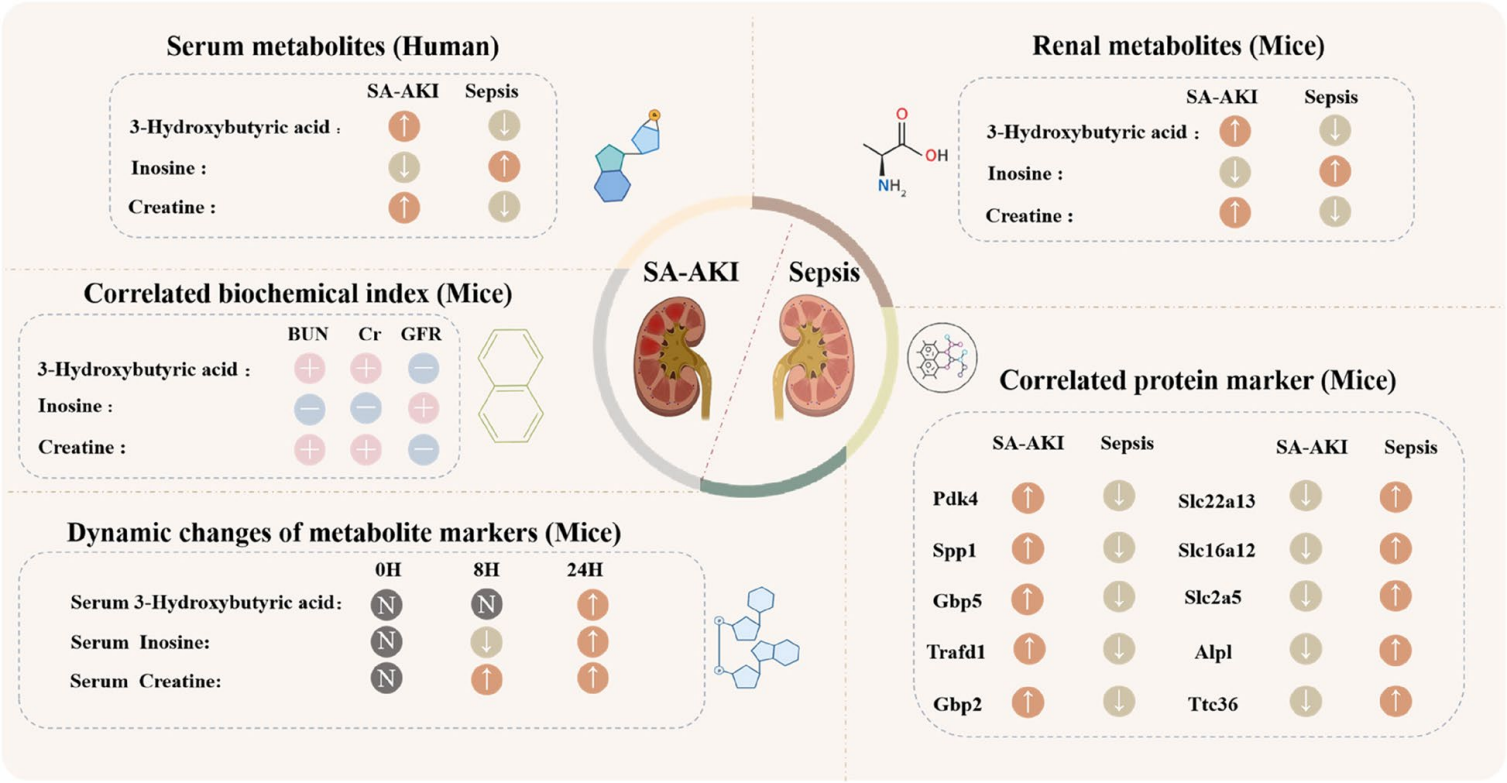
Characterization of core metabolites at multiple omics levels. The main molecular changes of mice renal metabolomics, mice renal proteomics, mice plasma metabolomics, mice biochemical indicators, and clinical plasma targeted metabolomics were summarized. Arrows represent up- or down-regulation of molecules in the SA-AKI group in comparison to the sepsis group. “ ± ” symbol indicates a positive or negative correlation between the core metabolite changes and biochemical indicators

By integrating renal metabolomics and proteomics, we constructed a multi-omics molecular network of SA-AKI and identified five core metabolites associated with SA-AKI, i.e., 3-hydroxybutyric acid, creatine, inosine, 3-hydroxymethylglutaric acid and myristic acid. Subsequently, we performed a time series analysis of serum from SA-AKI mice to assess the potential of core metabolites as biomarkers for the early diagnosis of SA-AKI. Eventually, we conducted a SA-AKI diagnostic model, IC3, using serum-targeted quantitative metabolomics for SA-AKI patients with an AUC of 0.90. The major molecules associated with SA-AKI are summarized in Fig. 7.

This study employed several cutting-edge technologies, including GC-TOF–MS, 4D label-free analysis, and real-time glomerular filtration rate (GFR) monitoring technology, to enable the high-dimension and in-depth investigation of SA-AKI. 3-Hydroxybutyric acid, inosine, and creatine were identified as three core metabolites in the early stage of SA-AKI. Previous studies have shown that 3-hydroxybutyric acid is one of the ketone bodies synthesized by the liver during fasting [37, 38]. In patients with sepsis or trauma, an increase in 3-hydroxybutyric acid levels was observed [39–41], suggesting that these patients may have entered a state of ketosis due to insufficient glucose supply in the blood. In addition, the activity of inosine is highly consistent with GRF, the same conclusion was also found in previous studies [42, 43]. For instance, Szabó et al. observed that additional inosine supplementation can reduce creatinine and BUN in cecal ligation and puncture (CLP) mice [44], which potentially ameliorates acute inflammation through modulation of the TLR4/NF-ΚB signaling pathway [45]. Furthermore, urinary creatine levels elevated in several AKI mice models induced by multiple drugs, such as polymyxin B [46], aristolochic acid [47], and gentamicin [48], which may be due to oxidative modification of renal mitochondria or cellular lysosomal creatine kinase [49, 50]. We observed that creatine continued to rise during the progression of SA-AKI, and this change affects several metabolic pathways, such as arginine and proline metabolism. Although creatine changes were not significant in female patients, creatine has the potential to be a valuable biomarker for renal injury. Drawing upon the possible significance of inosine, creatine, and 3-hydroxybutyric acid, this study formulated the IC3 model to early prediction of SA-AKI in patients with sepsis.

These three metabolites in the IC3 model have been separately used in clinic. For example, creatine is a sports supplement used to increase muscle strength, but some studies have reported that taking creatine may cause interstitial nephritis [51]. In addition, inosine supplementation effectively elevates human serum uric acid levels without compromising renal function [52–54]. However, these three core metabolites have not yet been utilized in the early screening and diagnosis of SA-AKI. We noted that early detection of 3-hydroxybutyric acid, creatine, and inosine in patients with sepsis can predict the occurrence of SA-AKI, and further found that the nomogram constructed via the IC3 model was positively correlated with APACHE II and SOFA scores. IC3 model is instrumental in the early identification of high-risk individuals among patients with sepsis who are potentially susceptible to developing SA-AKI, which can also serve as a guiding tool to assist medical care providers in offering early intervention [55]. Moreover, for SA-AKI patients with higher IC3 scores, early initiation of treatment such as renal replacement therapy (RRT) can be considered to avoid persistent renal damage and reduce mortality [56, 57].

Current researches on SA-AKI lack temporal sensitivity [57]. To this end, we performed a time series analysis of metabolites to determine the optimal time point for diagnosing SA-AKI. Because of the delay between increased creatinine concentration and the occurrence of AKI, interventions for AKI typically are initiated during the maintenance phase of renal injury [58]. Early screening for SA-AKI can be conducted within the first 24 h to promptly identify high-risk individuals, mitigate nephrotoxic drug use, and adjust dosages according to renal function; Simultaneous injecting vasoactive drugs to maintain mean arterial pressure at least 65 mmHg [59] to prevent irreversible structural renal damage. Moreover, in contrast to mice metabolomics or proteomics studies [13, 14], our work innovatively integrated three different omics including mice renal metabolomics, renal proteomics, serum time series metabolomics, and population-targeted quantitative metabolomics to identify SA-AKI biomarkers. This multidimensional strategy greatly enhances the precision with identified biomarkers and allows us to track the dynamic change of these metabolites during the development of SA-AKI.

LPS (*i.p.*) is a classic sepsis mice model [60, 61]. Inflammatory pathways are activated, accompanied by increased mitochondrial dysfunction and disrupted energy metabolism, which further triggers metabolic reprogramming and programmed cell death in target organs, such as renal [62, 63]. This series of pathophysiological processes eventually led to the occurrence of AKI. Peng et al. found that SAP130 protein released after iron death activation can induce macrophage polarization, which further exacerbates the damage of the renal tubular [64]. On the other hand, previous studies used to apply serum creatinine, urea nitrogen, and renal pathologic score to determine whether sepsis mice were complicated with AKI [65–67]. On top of that, we combined GFR technology to successfully construct an early and stable LPS-induced SA-AKI model and double-verified it by molecular markers such as NGAL [23]. Therefore, we continued to use this technique to ensure the stability and reliability of the SA-AKI mice model.

Study limitations include the small clinical sample size and the single-center nature of participant recruitment, the potential confounders in the construction of IC3, such as medication usage, dietary habits, and lifestyle factors, only male mice were used in the experiments, and the lack of molecular mechanisms underlying these metabolic variations. Consistent with previous studies that utilized male mice [68, 69], these animals exhibit minimal individual variation and greater stability across body size, metabolic rate, and behavioral physiological responses. On the other hand, female mice experience fluctuations in estrogens and other hormones [70], which have the potential to interfere with metabolic processes and consequently influence the experimental data interpretability. Although only 56 samples were used in this study for model construction, we demonstrated the reliability of our model with multiple experimental designs, including reliable SA-AKI mice models, multi-omics of renal tissue, and serum time series metabolomics. On top of that, we are planning to expand the collection of more diverse clinical samples and conduct multi-center studies to keep updating the current model.

## Conclusions

Through renal multi-omics network analyses and serum time series studies in SA-AKI mice, core metabolites were identified as potential SA-AKI diagnostic biomarkers. Serum quantitative metabolomic analysis of a clinical cohort was performed to develop a metabolite-based diagnostic model, IC3, enabling the early detection of SA-AKI. The current results are only preliminary findings, and prospective validation in a more diverse clinical population is needed to verify the accuracy and reliability of the IC3 model.

## Supplementary Information

Additional file 1. Detailed metabolomics, proteomics sequencing methods and data quality control methods.

Additional file 2. Biochemical index and renal metabolomics data in mice. Table S1. Biochemical index between SA-AKI mice and sepsis mice. Table S2. Renal metabolomics data. Table S3. Renal metabolomics annotation.

Additional file 3. Renal proteomics analysis. Table S1. Differential renal proteins between SA-AKI and sepsis in mice. Figure S1. Mice renal proteins and enrichment analysis. a) Abundance distributions of mice renal metabolomics and proteomics. b) Heatmap showing the correlation between renal proteins and the three biochemical indicators. Orange indicates a positive correlation and blue indicates a negative correlation. c-e) Gene Ontology Enrichment Analysis for renal differential proteins, including biological process (BP) (c); cell composition (CC) (d); molecular functional (MF) (e).

Additional file 4. The expression of five core metabolites in serum metabolomics. Table S1. Serum metabolomics in SA-AKI mice. Table S2. Quantitative serum metabolomics in patients with SA-AKI or sepsis. Figure S1. Distribution of 3-Hydroxybutyric acid, inosine and creatine in male and female patients. a-b) Scatter plots illustrating the correlation between inosine, myristic acid and BUN. c) Heatmap of 3-hydroxybutyric acid, creatine, and inosine between sepsis and SA-AKI after grouping by sex. d-f) Box plot of 3-hydroxybutyric acid, inosine and creatine between sepsis and SA-AKI in male group. g-i) Box plot of 3-hydroxybutyric acid, inosine and creatine between sepsis and SA-AKI in female group. Green represents sepsis and purple represents SA-AKI. *, ** and *** indicate *p* < 0.05, *p* < 0.01, and *p* < 0.001, respectively. Renal proteomics data has been deposited in the ProteomeXchange Consortium via the PRIDE (Proteomics Identifications) partner repository with the dataset identifier PXD057050.

## Abbreviations

SA-AKI: Sepsis-associated acute renal injury
ICU: Intensive care unit
NGAL: Neutrophil gelatinase-associated lipocalin
KIM-1: Kidney injury molecule-1
sTM: Soluble thrombomodulin
L-FABP: Liver-type fatty acid binding protein
TIMP-2: Tissue inhibitor of metalloproteinases-2
IGFBP-7: Insulin-like growth factor-binding protein 7
GFR: Glomerular filtration rate
BUN: Blood urea nitrogen
Cr: Creatinine
KDIGO: Kidney Disease Improving Global Outcomes
FDR: False discovery rate
FC: Fold change
OR: Odds ratio
ROC: Receiver operating characteristic
LR: Logistic regression
RF: Random forest
SVM: Support vector machine
XGB: Extreme gradient boosting
CLP: Cecal ligation and puncture
RRT: Renal replacement therapy
