# Correction: Metabolomics- and proteomics-based multi-omics integration reveals early metabolite alterations in sepsis-associated acute kidney injury

**DOI:** 10.1186/s12916-025-03980-9

**Published:** 2025-03-08

**Authors:** Pengfei Huang, Yanqi Liu, Yue Li, Yu Xin, Chuanchuan Nan, Yinghao Luo, Yating Feng, Nana Jin, Yahui Peng, Dawei Wang, Yang Zhou, Feiyu Luan, Xinran Wang, Xibo Wang, Hongxu Li, Yuxin Zhou, Weiting Zhang, Yuhan Liu, Mengyao Yuan, Yuxin Zhang, Yuchen Song, Yu Xiao, Lifeng Shen, Kaijiang Yu, Mingyan Zhao, Lixin Cheng, Changsong Wang

**Affiliations:** 1 Department of Critical Care Medicine, The First Affiliated Hospital of Harbin Medical University, Harbin, Heilongjiang 150001 China; 2 Department of Critical Care Medicine, First Affiliated Hospital of Southern, Shenzhen People’s Hospital, University of Science and Technology, Shenzhen, 518020 China; 3 Department of Critical Care Medicine, Second Affiliated Hospital of Harbin Medical University, Heilongjiang Province, Harbin, 150086 China; 4 Department of Critical Care Medicine, Harbin Medical University Cancer Hospital, Harbin, 150081 China; 5 Heilongjiang Provincial Key Laboratory of Critical Care Medicine, 23 Postal Street, Nangang District, Harbin, Heilongjiang 150001 China

**Correction** **:** **BMC Med 23, 79 (2025)**

**https://doi.org/10.1186/s12916-025-03920-7**

The authors wish to note corrections affecting citations and funding in the original article [1].

The following citations are affected and their suggested revisions noted accordingly:

• Citation Fig. 2c of Line 270 should instead read as Fig. 3c.
• Citation Fig. 3c of Line 288 should instead read as Fig. 3d.
• Citation Fig. 3d of Line 292 should instead read as Fig. 3e.
• Citation Fig. 3e for Line 296 should instead read as Fig. 3f.
