# Supplementary material for: Metabolomics- and proteomics-based multi-omics integration reveals early metabolite alterations in sepsis-associated acute kidney injury
Source: BMC Med. 2025 Feb 11;23:79. doi: 10.1186/s12916-025-03920-7 (PMC11818193; doi:10.1186/s12916-025-03920-7)
Supplement: Supplementary file 1 — Additional file 1. Detailed metabolomics, proteomics sequencing methods and data quality control methods. [file 12916_2025_3920_MOESM1_ESM.zip › Additional file 1/Supplementary Material.docx]

**S-AKI mice construction**

Post-modeling, the mice were euthanized following anesthesia with 150-200mg/kg pentobarbital sodium injected intraperitoneally. Blood was collected from the orbital sinus (0.2-0.3ml) into blood collection tubes and then placed on ice. The abdominal wall of the mice was cut open along the midline, the renal was removed and trimmed of unnecessary tissue, and then the renal tissues were placed in sterile cryotubes and stored in liquid nitrogen. The collected blood samples were centrifuged at 3000g for 15 minutes, after which the upper layer of plasma was extracted and stored at -80 degrees Celsius for testing.

On the other hand, we have adopted various strategies to minimize the influence of potential confounding factors during modeling. For example, by randomly assigning animals to lps (*i.p.*) groups or control groups, the balanced distribution of known and unknown confounding factors among groups is ensured. At the same time, the living environment of all mice is consistent, and the cage position of experimental animals is standardized to avoid potential bias caused by environmental differences. The SA-AKI mice construction and sample size for each experimental were referred to previous study (23). No adverse events occurred.

**Real-time fluorescence imaging technology**

The right abdominal hair of the mice was shaved, and a depilatory cream was subsequently used to clean the right abdominal area. The mice were anesthetized with isoflurane, and the optical device (from MediBeacon GmbH, Mannheim, Germany) was fixed to the shaved area. Once the mice regained consciousness, they were intravenously injected with mice sinistrin via the tail vein within 5 minutes to monitor fluorescence intensity, which was continuously observed for 1 hour. After removing the device under isoflurane anesthesia, the data was analyzed using MB Studio v. 22 (Medibeacon GmbH). During the monitoring process, the mice could move freely, with unrestricted access to water and food.

**Animal Serum Marker Testing**

Serum was used for creatinine and urea nitrogen testing. A reagent kit (Scr; Cat. No. c011-2-1; Nanjing Jiancheng Bioengineering Institute) was used to measure serum creatinine according to the manufacturer's instructions. An automatic biochemical analyzer (IDEEX) was used to determine blood urea nitrogen.

**Metabolomics**

**Gas Chromatography-Time of Flight Mass Spectrometry (GC-TOF-MS)**

Utilizing Gas Chromatography-Time of Flight Mass Spectrometry (GC-TOF-MS) (24) , we conducted an analysis of renal tissues from mice afflicted with sepsis and S-AKI, focusing on the dynamic shifts in small molecule metabolites due to external stimuli.

The metabolite extraction process entailed: mixing a 25±1 mg sample with 500 µl of a chilled methanol/chloroform mixture (3:1 ratio) and 2-chloro-L-phenylalanine (1 mg/ml) as an internal standard, in a 2 ml tube, and subjecting it to 30 seconds of vortexing. Subsequently, steel beads were introduced, and the sample underwent 4 minutes of grinding at 40 Hz, followed by thrice-repeated ultrasonic treatments of 5 minutes each in an ice-water bath. Post-centrifugation at 4°C for 15 minutes (12000 rpm, 13800×g, 8.6 cm radius), 200 µl of the supernatant was moved to a new tube. In preparing quality control (QC) samples, 80 µl from each sample were combined. Post-evaporation in a vacuum concentrator, 30 µl of methoxyamine hydrochloride (20 mg/ml in pyridine) was added, incubated at 80°C for 30 minutes, then derivatized with 40 µl of BSTFA reagent (1% TMCS, v/v) at 70°C for 1.5 hours. Upon cooling to room temperature, 5 µl of FAMEs (in chloroform) was added to the QC samples. Finally, an Agilent 7890 Gas Chromatograph, coupled with a Time of Flight Mass Spectrometer, was employed for analyzing all samples.

The system utilized a DB-5MS capillary column. 1 μL aliquot of sample was injected in splitless mode. Helium was used as the carrier gas, the front inlet purge flow was 3 mL min −1 , and the gas flow rate through the column was 1 mL min −1 . The initial temperature was kept at 50 °C for 1 min, then raised to 310 °C at a rate of 10 °Cmin−1 , then kept for 8 min at 310 °C. The injection, transfer line, and ion source temperatures were 280, 280and 250 °C, respectively. The energy was -70 eV in electron impact mode. The mass spectrometry data were acquired in full-scan mode with the m/z range of 50-500 at a rate of 12.5spectra per second after a solvent delay of 6.27 min.

The raw data analysis encompassing peak extraction, baseline adjustment, deconvolution, alignment, and integration, was performed using Chroma TOF software (V 4.3x, LECO) (25). Metabolites identification was enabled by the LECO-Fiehn Rtx5 database, through mtching of mass spectra and retention indices. Peaks identified in fewer than half of the QC samples or those with an RSD exceeding 30% in QC samples were excluded . Further data processing for each peak involved outlier filtering based on quartile data, imputation of missing values using the minimum value/2 method, and normalization against internal standards. A threshold was set to exclude any peak with a minimum value below 1E-05, thus ensuring data accuracy and reliability.

**Ultra-performance liquid chromatography(UPLC/MS)**

From S-AKI mice plasma, 16 samples were extracted and divided into four groups for plasma metabolomics analysis using the UPLC-MS/MS detection platform (26).

Extraction of hydrophilic compounds: the plasma samples were thawed from -80°C freezer and vortexed for 10 seconds. For hydrophilic compounds, 50μL of the sample was mixed with 300μL of acetonitrile: methanol extraction solution (1:4, v/v), internal standards were added, and the mixture was placed in a 2 mL microcentrifuge tube. After shaking for 3 minutes, it was centrifuged at 12000rpm for 10 minutes at 4°C, and 200μL of the supernatant was collected and stored at -20°C for 30 minutes. Then, it was centrifuged for an additional 3 minutes, and 180μL of the supernatant was transferred to an LC-MS sample vial.

Extraction of hydrophobic compounds: 50μL of the sample was mixed with 1mL of MTBE: methanol extraction solvent (3:1, v/v) containing internal standards. After 15 minutes of mixing, 200μL of water was added, followed by 1 minute of mixing. The mixture was then centrifuged for 10 minutes, and 200μL of the organic layer was collected. After vacuum concentration, it was dissolved in 200μL of acetonitrile: isopropanol reconstitution solution (1:1, v/v) for LC-MS/MS analysis.

UPLC Conditions of hydrophilic compounds: The sample extracts used an LC-ESI-MS/MS system, including UPLC (ExionLC AD) and MS (QTRAP® system). Key parameters included the use of a Waters ACQUITY UPLC HSS T3 C18 column (1.8 μm, 2.1 mm × 100 mm) with a column temperature set at 40°C and a flow rate of 0.4 mL/min. The injection volume was 2 μL, and the solvent system consisted of water with 0.1% formic acid and acetonitrile. Gradient program, 95:5 V/V at 0 min, 10:90 V/V at 11.0 min,10:90 V/V at 12.0 min, 95:5 V/V at 12.1 min, 95:5 V/V at 14.0 min.

UPLC Conditions of hydrophobic compounds: The sample extracts were analyzed using an LC-ESI-MS/MS system (UPLC, ExionLC AD，MS, QTRAP® System). The analytical conditions were as follows, UPLC: column, Thermo Accucore™ C30 (2.6 μm, 2.1 mm*100 mm i.d.). Solvent system, A: acetonitrile/water (60/40,V/V, 0.1% formic acid, 10 mmol/L ammonium formate), B: acetonitrile/isopropanol (10/90 V/V, 0.1% formic acid, 10 mmol/L ammonium formate); The gradient program started at A/B (80:20, v/v) at 0 minutes, 70:30 V/V at 2.0 min, 40:60 V/V at 4 min, 15:85 V/V at 9 min, changed to 10:90 at 14 minutes, further changed to 5:95 at 15.5 minutes and 17.3 minutes, and returned to 80:20 at 20 minutes. The flow rate was set at 0.35 mL/min, the temperature was controlled at 45°C, and the injection volume was 2 μL. The effluent was alternatively connected to an ESI-triple quadrupole-linear ion trap (QTRAP)-MS.

QTOF-MS/MS : After analysis, the effluent was directed into the ESI Triple Quadrupole Linear Ion Trap (QTRAP) mass spectrometer for further detection and analysis. The Triple TOF mass spectrometer was used because it could obtain MS/MS spectra in information-dependent acquisition (IDA) mode during liquid chromatography/mass spectrometry (LC/MS) experiments. In this mode, the data acquisition software (TripleTOF 6600, AB SCIEX) continuously evaluated the full-scan MS data it collected and triggered the collection of MS/MS spectra based on preset criteria. In each cycle, 12 precursor ions with intensities greater than 100 were selected for collision-induced dissociation (CID) at 30V (12 MS/MS events, each with a product ion accumulation time of 50 milliseconds). The conditions for electrospray ionization (ESI) were as follows: ion source gas 1 at 50 psi, ion source gas 2 at 50 psi, curtain gas at 25 psi, source temperature at 500°C, and ion spray voltage floating (ISVF) at 5500V (positive mode) or -4500V (negative mode). Subsequently, hydrophilic and hydrophobic compounds were analyzed using the ESI-Q TRAP-MS/MS system.

ESI-Q TRAP-MS/MS of hydrophilic compounds: LIT and triple quadrupole (QQQ) scans were acquired on a triple quadrupole-linear ion trap mass spectrometer (QTRAP), QTRAP® LC-MS/MS System, equipped with an ESI Turbo Ion-Spray interface, operating in positive and negative ion mode and controlled by Analyst 1.6.3 software (Sciex). The ESI source parameters included a source temperature of 500°C, ion spray voltage of 5500V (positive) and -4500V (negative), ion source gas I, gas II, and curtain gas set at 55, 60, and 25.0 psi, respectively; collision-activated dissociation (CAD) was set to high. Instrument calibration and mass calibration were performed using 10 and 100 µM polypropylene glycol solutions. Based on the metabolites eluted during this period, a specific set of multiple reaction monitoring (MRM) transitions was monitored.

ESI-Q TRAP-MS/MS of hydrophobic compounds: LIT and triple quadrupole (QQQ) scans were acquired on a triple quadrupole-linear ion trap mass spectrometer (QTRAP), QTRAP® LC-MS/MS System, equipped with an ESI Turbo Ion-Spray interface, operating in positive and negative ion mode and controlled by Analyst 1.6.3 software (Sciex). The ESI source operation parameters were as follows: the ion source was Turbo Spray, source temperature remained at 500°C, ion spray voltage was 5500V (positive) and -4500V (negative), ion source gas 1, gas 2, and curtain gas were set at 45, 55, and 35 psi, respectively; collision-activated dissociation (CAD) was set to medium (27) . The collision gas (CAD) was medium. Instrument tuning and mass calibration were performed with 10 and 100 μmol/L polypropylene glycol solutions in QQQ and LIT modes, respectively. QQQ scans were acquired as MRM experiments with collision gas (nitrogen) set to 5 psi. and the declustering potential (DP) and collision energy (CE) were optimized for each MRM transition. A specific set of MRM transitions were monitored for each period according to the metabolites eluted within this period.

**T500 Serum Kit**

Targeted quantitative and qualitative analysis (29) was conducted on 56 patient samples recruited for clinical research using LC-MS/MS methodology. Five differential metabolites related to sepsis and SA-AKI were first identified in mice experiments, but only three out of them, including 3-hydroxybutyric acid, inosine and creatine were detected in the T500 results. So, these three metabolites were used for the subsequent analysis.

Sample preparation and extraction: After the samples were thawed, they were vortex-mixed for 10 seconds. Fifty microliters of the sample were transferred to a centrifuge tube, mixed with 250 microliters of a 20% acetonitrile/methanol (purchased from Merck (Darmstadt, Germany)) mixture, vortex-mixed for 3 minutes, and then centrifuged at 12,000 rpm for 10 minutes at 4°C. Two hundred and fifty microliters of the supernatant were transferred to a new centrifuge tube and placed in a -20°C freezer for 30 minutes. Subsequently, it was centrifuged again at 12,000 rpm for 10 minutes at 4°C. After centrifugation, 180 microliters of the supernatant were transferred using a protein precipitation plate for further liquid chromatography-mass spectrometry (LC-MS) analysis.

UPLC Conditions: Sample extracts were analyzed using an LC-ESI-MS/MS system (UPLC, ExionLC AD; MS, QTRAP 6500+ system) (28). T3 method：HPLC: column, Waters ACQUITY UPLC HSS T3 C18 (100 mm×2.1 mm i.d.ˈ1.8 µm); solvent system, water with 0.05% formic acid (A), acetonitrile with 0.05% formic acid (B). The gradient was started at 5% B (0 min), increased to 95% B (8-9.5 min), finaly ramped back to 5% B (9.6-12 min); flow rate, 0.35 mL/min; temperature, 40°C; injection volume: 2 μL. Amide method: HPLC: column, ACQUITY UPLC BEH Amide (i.d.2.1×100 mm, 1.7 μm); solvent system, water with 10mM Ammonium acetate and 0.3% Ammonium hydroxide (A), 90% acetonitrile/water (V/V)(B); The gradient was started at 95% B (0-1.2 min), decreased to 70% B (8 min),50% B (9-11 min), finaly ramped back to 95% B (11.1-15 min); flow rate, 0.4 mL/min; temperature, 40°C; injection volume: 2 μL.

ESI-MS/MS Conditions: Linear ion trap (LIT) and triple quadrupole (QQQ) scans were acquired on a triple quadrupole-linear ion trap mass spectrometer (QTRAP), QTRAP® 6500+ LC-MS/MS System, equipped with an ESI Turbo Ion-Spray interface, operating in both positive and negative ion mode and controlled by Analyst 1.6.3 software (Sciex). The ESI source operation parameters were as follows: ion source, ESI+/-; source temperature 550 ℃; ion spray voltage (IS) 5500 V（Positive）,-4500 V(Negative); curtain gas (CUR) was set at 35 psi, respectively. Metabolites were analyzed using scheduled multiple reaction monitoring (MRM). Data acquisitions were performed using Analyst 1.6.3 software (Sciex). Multiquant 3.0.3 software (Sciex) was used to quantify all metabolites. Mass spectrometer parameters including the declustering potentials (DP) and collision energies (CE) for individual MRM transitions were done with further DP and CE optimization. A specific set of MRM transitions were monitored for each period according to the metabolites eluted within this period.

Detection of targeted metabolites: All those metobolites were detected based on the AB Sciex QTRAP 6500 LC-MS/MS platform.

**Proteomics**

Protein Extraction: Firstly, mice renal tissues were retrieved from -80°C storage conditions and placed in a pre-cooled bowl with liquid nitrogen, where they were ground into powder. Subsequently, each sample was mixed with four times the volume of powder lysis buffer (containing 8 M urea and 1% protease inhibitor) and underwent three rounds of sonication while on ice. Following that, centrifugation at 4°C and 12,000 g for 10 minutes was performed to remove cell debris, and the supernatant was collected for protein concentration determination using a BCA assay.

Enzymatic Digestion: Take an equal amount of protein from each sample for enzymatic hydrolysis, and adjust the volume to the same with lysis buffer. Then slowly add TCA at a final concentration of 20%, vortex to mix, and precipitate at 4°C for 2h. Then centrifuge at 4500g for 5min, discard the supernatant, and wash the precipitate with pre-cooled acetone 2-3 times. After drying the precipitate, add TEAB with a final concentration of 200 mM, break up the precipitate by ultrasound, add trypsin at a ratio of 1:50 (protease: protein, m/m), and enzymatic hydrolysis overnight. Then add dithiothreitol (DTT) to a final concentration of 5 mM, and reduce at 56°C for 30 min. Finally, add iodoacetamide (IAA) to a final concentration of 11 mM and incubate at room temperature in the dark for 15 min.

Liquid Chromatography-Mass Spectrometry (LC-MS) Analysis: Peptides were dissolved in mobile phase A (0.1% formic acid and 2% acetonitrile in water) and separated using an EASY-nLC 1200 ultra-high-performance liquid chromatography system (30). The gradient elution followed this program: 0-68 minutes, 6%~23% B; 68-82 minutes, 23%~32% B; 82-86 minutes, 32%~80% B; 86-90 minutes, 80% B, with a flow rate of 500 nL/min. Peptides were ionized via the NSI source and analyzed on an Orbitrap Exploris™ 480 mass spectrometer. Mass spectrometer parameters were set as follows: ion source voltage 2.3 kV, FAIMS compensation voltages -45 V and -65 V. Both the peptide precursor ions and their secondary fragments were detected and analyzed using a high-resolution Orbitrap. The primary mass spectrometry scan range was set to 400-1200 m/z, and the scan resolution was set to 60000; the secondary mass spectrometry scan range had a fixed starting point of 110 m/z, the secondary scan resolution was set to 15000, and TurboTMT was set to Off. The data acquisition mode used a data-dependent scan (DDA) program, which is, after the primary scan, the top 25 peptide precursor ions with the highest signal intensity were selected to enter the HCD collision cell in turn and use 27% fragmentation energy for fragmentation, and the secondary mass spectrometry analysis was also performed in turn. In order to improve the effective utilization of the mass spectrometer, the automatic gain control (AGC) was set to 100%, the signal threshold was set to 5E4 ions/s, the maximum injection time was set to Auto, and the dynamic exclusion time of the tandem mass spectrometry scan was set to 20 s to avoid repeated scanning of the precursor ion.

Database search: The resulting MS/MS data were processed using MaxQuant search engine (v.1.6.15.0). Tandem mass spectra were searched against the human SwissProt database (20422 entries) concatenated with reverse decoy database. Trypsin/P was specified as cleavage enzyme allowing up to 2 missing cleavages. The mass tolerance for precursor ions was set as 20 ppm in first search and 5 ppm in main search, and the mass tolerance for fragment ions was set as 0.02 Da. Carbamidomethyl on Cys was specified as fixed modification, and acetylation on protein N-terminal and oxidation on Met were specified as variable modifications. FDR was adjusted to<1%.

**Metabolomics and Proteomics Data Quality Control**

Gas Chromatography-Mass Spectrometry (GC-MS) was utilized for metabolomic profiling of 16 renal samples from mice. This process led to the detection of 467 renal metabolites. A minimum intensity threshold of 1E-05 was set to filter out weaker signals, resulting in the identification of 209 metabolites. Among these, annotations for 120 metabolites were available in existing biological databases. When analyzing 16 mice renal samples, a significantly low metabolic activity was observed in sepsis SK4 sample (Additional file 2: **Figure S1a**), excluding this sample from subsequent statistical analysis. For the GC-Quad FiehnLib library, we named derivatives by increasing numbers according to the retention index , and for derivative metabolites, metabolites with larger fold change were retained.

Untargeted proteomics sequencing was conducted on renal tissue samples from 12 sepsis and S-AKI mice, identifying 5782 proteins. Following stringent quality control measures, which included the exclusion of proteins with more than 50% missing values, a total of 5119 proteins were retained for further analysis.
