# Supplementary figures and images for: Metabolomics- and proteomics-based multi-omics integration reveals early metabolite alterations in sepsis-associated acute kidney injury
Source: BMC Med. 2025 Feb 11;23:79. doi: 10.1186/s12916-025-03920-7 (PMC11818193; doi:10.1186/s12916-025-03920-7)

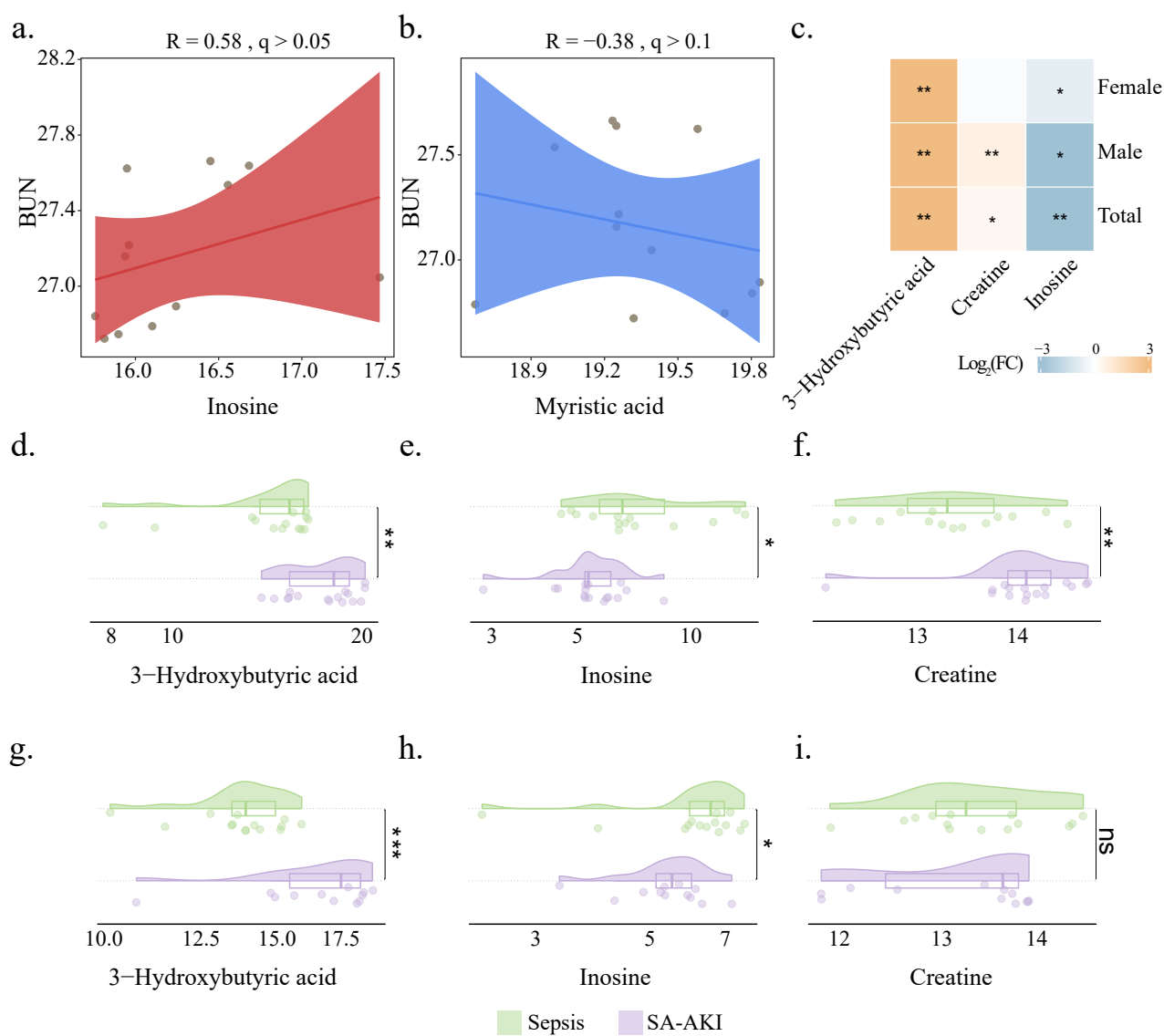

Supplement: Supplementary file 4 — Additional file 4. The expression of five core metabolites in serum metabolomics. Table S1. Serum metabolomics in SA-AKI mice. Table S2. Quantitative serum metabolomics in patients with SA-AKI or sepsis. Figure S1. Distribution of 3-Hydroxybutyric acid, inosine and creatine in male and female patients. a-b) Scatter plots illustrating the correlation between inosine, myristic acid and BUN. c) Heatmap of 3-hydroxybutyric acid, creatine, and inosine between sepsis and SA-AKI after grouping by sex. d-f) Box plot of 3-hydroxybutyric acid, inosine and creatine between sepsis and SA-AKI in male group. g-i) Box plot of 3-hydroxybutyric acid, inosine and creatine between sepsis and SA-AKI in female group. Green represents sepsis and purple represents SA-AKI. *, ** and *** indicate p < 0.05, p < 0.01, and p < 0.001, respectively. Renal proteomics data has been deposited in the ProteomeXchange Consortium via the PRIDE (Proteomics Identifications) partner repository with the dataset identifier PXD057050. [file 12916_2025_3920_MOESM4_ESM.zip › Additional file 4/Figure S1.pdf]
