# Supplementary material for: Metabolomics- and proteomics-based multi-omics integration reveals early metabolite alterations in sepsis-associated acute kidney injury
Source: BMC Med. 2025 Feb 11;23:79. doi: 10.1186/s12916-025-03920-7 (PMC11818193; doi:10.1186/s12916-025-03920-7)
Supplement: Supplementary file 3 — Additional file 3. Renal proteomics analysis. Table S1. Differential renal proteins between SA-AKI and sepsis in mice. Figure S1. Mice renal proteins and enrichment analysis. a) Abundance distributions of mice renal metabolomics and proteomics. b) Heatmap showing the correlation between renal proteins and the three biochemical indicators. Orange indicates a positive correlation and blue indicates a negative correlation. c-e) Gene Ontology Enrichment Analysis for renal differential proteins, including biological process (BP) (c); cell composition (CC) (d); molecular functional (MF) (e). [file Figure S1.pdf › Additional file 3/Figure S1.pdf]

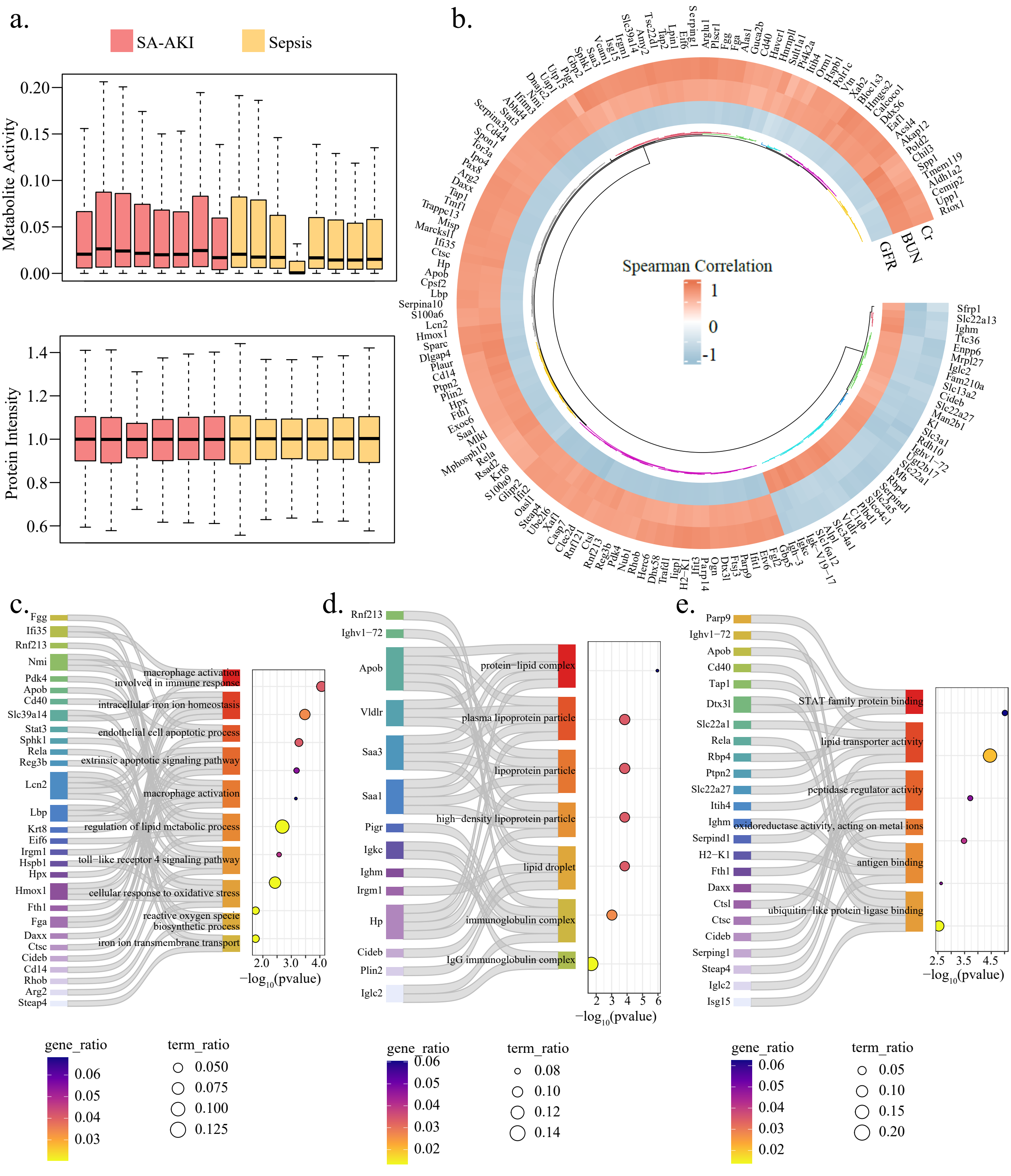

a.
SA-AKI
Sepsis
Metabolite Activity
0.20
0.15
0.10
0.05
0.00
Protein Intensity
1.4
1.2
1.0
0.8
0.6
b.
Spearman Correlation
1
0
-1
Cr
BUN
GFR
Serping1 Arglu1 Plscr1 Fgg Fga Alas1 Guca2b Cd40 Havcr1 Hrmpll Sult1a1 Pi4k2a Itih4 Orm1 Hspb1 Polr1c Vtn Xab2 Bloc1s3 Hmgcs2 Calcoco1 Ddx56 Eaf1 Acsl4 Akap12 Pold2 Chil3 Spp1 Tmem119 Aldh1a2 Cemip2 Upp1 Riox1
Sfrp1 Slc22a13 Ighm Ttc36 Enpp6 Mrpl27 Iglc2 Fam210a Slc13a2 Cideb Slc22a27 Man2b1 Kl Slc3a1 Rdh10 Ighv1−72 Ugt2b17 Slc22a1 Mb Rbp4 Serpind1 Slc2a5 Slc4c1 Plbd1 C1qb Vldlr Slc34a1 Alpl Slc16a12 Igk−V19−17 Igkc Igh−3 Gbp5 Fgl2 Etv6 Ifit1 Parp9 Ftsj3 Dtx3l Ogn Parp14 Ifit3 H2−K1 Iigp1 Trafd1 Dhx58 Herc6 Rhob Nub1 Pdk4 Reg3b Rnf213 Ctsl Rnf121 Clec2d Casp7 Xaf1 Ube2l6 Steap4 Oasl1 Ifit2 Glipr2 S100a9 Krt8 Rsad2 Rela Mphosph10 Mlkl Saa1 Exoc6 Fth1 Hpx Plin2 Ptpn2 Cd14 Plaur Dlgap4 Sparc Hmox1 Lcn2 S100a6 Serpina10 Lbp Cpsf2 Apob Hp Ctsc Ifi35 Marcksl1 Misp Trappc13 Tmf1 Tap1 Daxx Arg2 Pax8 Ipo4 Tor3a Spon1 Cd44 Serpina3n Stat3 Abhd4 Ifitm3 Nmi Dnajc3 Uap1 Usp1 Pigr Gbp2 Sphk1 Saa3 Vcam1 Isg15 Irgm1 Slc39a14 Amy2 Tsc22d1 Tap2 Lpin1 Eif6
c.
Fgg
Ifi35
Rnf213
Nmi
Pdk4
Apob
Cd40
Slc39a14
Stat3
Sphk1
Rela
Reg3b
Lcn2
Lbp
Krt8
Eif6
Irgm1
Hspb1
Hpx
Hmox1
Fth1
Fga
Daxx
Ctsc
Cideb
Cd14
Rhob
Arg2
Steap4
macrophage activation involved in immune response
intracellular iron ion homeostasis
endothelial cell apoptotic process
extrinsic apoptotic signaling pathway
macrophage activation
regulation of lipid metabolic process
toll−like receptor 4 signaling pathway
cellular response to oxidative stress
reactive oxygen specie biosynthetic process
iron ion transmembrane transport
2.0
3.0
4.0
−log10(pvalue)
gene_ratio
0.06
0.05
0.04
0.03
term_ratio
0.050
0.075
0.100
0.125
d.
Rnf213
Ighv1−72
Apob
Vldlr
Saa3
Saa1
Pigr
Igkc
Ighm
Irgm1
Hp
Cideb
Plin2
Iglc2
protein−lipid complex
plasma lipoprotein particle
lipoprotein particle
high−density lipoprotein particle
lipid droplet
immunoglobulin complex
IgG immunoglobulin complex
2
3
4
5
6
−log10(pvalue)
gene_ratio
0.06
0.05
0.04
0.03
0.02
term_ratio
0.08
0.10
0.12
0.14
e.
Parp9
Ighv1−72
Apob
Cd40
Tap1
Dtx3l
Slc22a1
Rela
Rbp4
Ptpn2
Slc22a27
Itih4
Ighm
Serpind1
H2−K1
Fth1
Daxx
Ctsl
Ctsc
Cideb
Serping1
Steap4
Iglc2
Isg15
STAT family protein binding
lipid transporter activity
peptidase regulator activity
oxidoreductase activity, acting on metal ions
antigen binding
ubiquitin−like protein ligase binding
2.5
3.5
4.5
−log10(pvalue)
gene_ratio
0.06
0.05
0.04
0.03
0.02
term_ratio
0.05
0.10
0.15
0.20
